# Anorectal malignant mucosal melanoma

**DOI:** 10.4314/gmj.v56i4.12

**Published:** 2022-12

**Authors:** Antoinette A Bediako-Bowan, Hafisatu Gbadamosi, Hannah N G Ayettey, Philemon K Kumassah, Nicholas Aperkor, Selorm Dake, Uduak-Abasi Una, Joojo Nyamekye-Baidoo, Jonathan C B Dakubo

**Affiliations:** 1 Department of Surgery, University of Ghana Medical School, College of Health Sciences, University of Ghana, Korle Bu Campus, P. O. Box 4236, Accra; 2 Department of Surgery, Korle Bu Teaching Hospital, P. O. Box 77, Accra; 3 Department of Radiology, Korle Bu Teaching Hospital, P. O. Box 77, Accra; 4 National Radiotherapy Oncology and Nuclear Medicine Centre, Korle Bu Teaching Hospital, P. O. Box 77, Accra

**Keywords:** Anorectal, Malignant, Mucosal Melanoma, Surgical resection, Ghana

## Abstract

Anorectal mucosal melanoma (AMM) is a rare, aggressive malignancy. The symptoms of AMM mimic common benign conditions in the anus, such as haemorrhoids; hence diagnosis is often made late, a third of patients having metastasis at first presentation. Surgical resection remains the standard of treatment, and adjuvant therapy is varied, including immunotherapy, brachytherapy, and chemotherapy. The prognosis is poor, with a 5-year survival of 20%. A 65year old woman presented with a five-year history of symptoms suggestive of haemorrhoids and was diagnosed with a malignant anorectal mucosal melanoma after symptoms worsened and further investigation **was** performed. She underwent surgical resection and is currently receiving adjuvant therapy. The prognosis of AMM, the lack of consensus on the treatment regimen to dat**e,** and the need for a high index of suspicion for early diagnosis **are discussed**.

**Funding:**

None declared

## Introduction

Anorectal mucosal melanoma (AMM) is a rare malignancy. It accounts for 0.05% of all anorectal cancers.^1^ It is an aggressive neoplasm with a 5-year survival rate of 10%-20%, has a higher prevalence in patients above the sixth decade of life, and has a male to female ratio of 1:1.7.^1^–^3^ AMM commonly presents with bleeding per rectum, pain, a feeling of a mass in the anus, associated tenesmus, and constipation. Diagnosis is incidental and often made late due to nonspecific symptoms which mimic other benign conditions such as haemorrhoids, polyps, or other benign anorectal pathology.^4^ Surgical resection remains the standard for treatment of AMM, which could be a wide local excision (WLE) or an abdominal perineal resection (APR).^3^ We present a case of anorectal malignant mucosal melanoma treated with abdominoperineal resection.

## Case Report

A sixty-five-year-old woman presented to the surgical department of the Korle Bu teaching hospital with bleeding per rectum. She had a 5-year history of pruritus ani, two years of anal pain on defecation, relieved with suppository analgesics, and bleeding on defecation. She was managed for haemorrhoids conservatively by a general practitioner. Four months before the presentation, her bowel habits changed from once/twice a day to six times a day, with associated tenesmus and faecal incontinence. She had a 7-month history of a protruding mass from the anus, which had increased in size and was manually reducible, but with time had affected her ability to sit. She had a 15-year history of well-controlled hypertension and no other comorbidities.

**Figure 1:**
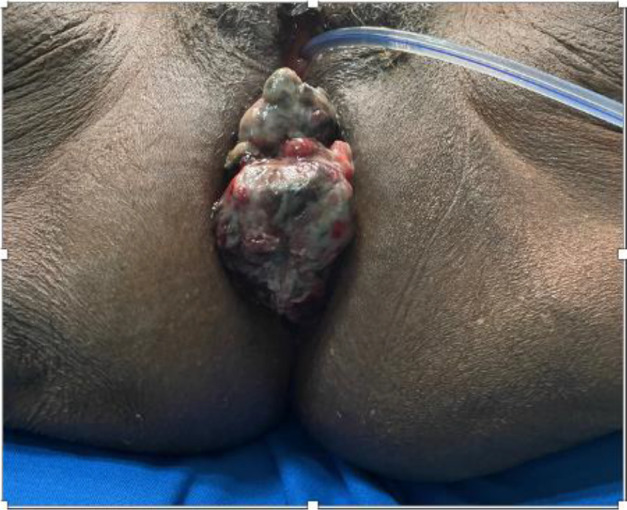
Picture of the anorectal lesion. Anorectal lesion protruding from anus (urethral catheter in situ)

On physical examination, there was a prolapsed 6cm lesion which was palpated 1 cm from the anal verge, extending clockwise from 11 o'clock through to 3 o'clock with a 4cm extension cranially into the anorectal canal (Figure 1). A complete colonoscopy showed the anorectal lesion and a single sessile polyp in the caecum. The anorectal lesion biopsy revealed a malignant mucosal melanoma and the caecal polyp, a tubulovillous adenoma with no malignant cells.

**Figure 2:**
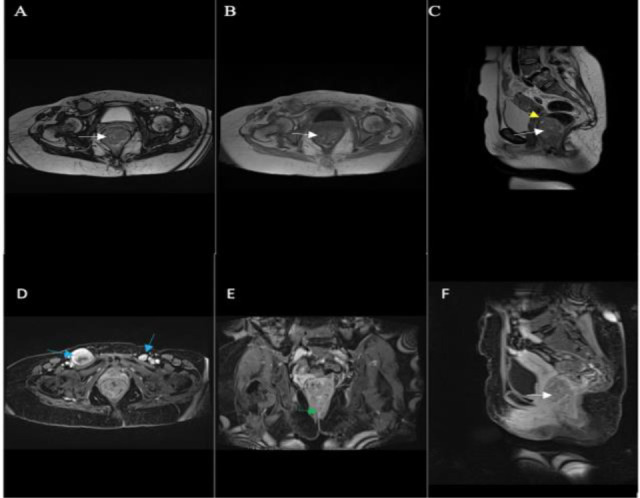
Magnetic resonance imaging (MRI) findings of anorectal mucosal melanoma of the patient. A-C; Axial T2 weighted; Axial T1 weighted; Sagittal T2 weighted; D-F Axial, coronal, and sagittal T1 weighted, fat-suppressed with contrast images, demonstrates a heterogeneously enhancing polypoid anorectal mass (white arrows) which shows prominent T2 hypointense and T1 hyperintense signal; it does not breach the rectal wall (yellow arrow). It extends to the anal verge (green arrow) with possible sphincteric muscle involvement and enlarged bilateral inguinal lymph nodes (blue arrows).

Staging with magnetic resonance imaging of the pelvis and abdomen showed a heterogeneously enhancing polypoid tumour along the left anterolateral wall of the lower rectum and anal canal. This showed prominent T2 hypointense and T1 hyperintense signals suggesting the presence of melanin or hemorrhage; it extended to the anal verge and spanned 66mm craniocaudally with luminal compromise. There was no infiltration of the mesorectal fat or fascia with no extraluminal extension. Multiple prominent mesorectal lymph nodes measured ∼ 6.9mm on average; enlarged bilateral iliac chain and inguinal lymph nodes. There was a large dominant right-sided lymph node seen, which measured approximately 40mm in short-axis diameter. Other abdominal organs were unremarkable (Figure 2). A chest x-ray showed regular lung fields.

**Figure 3:**
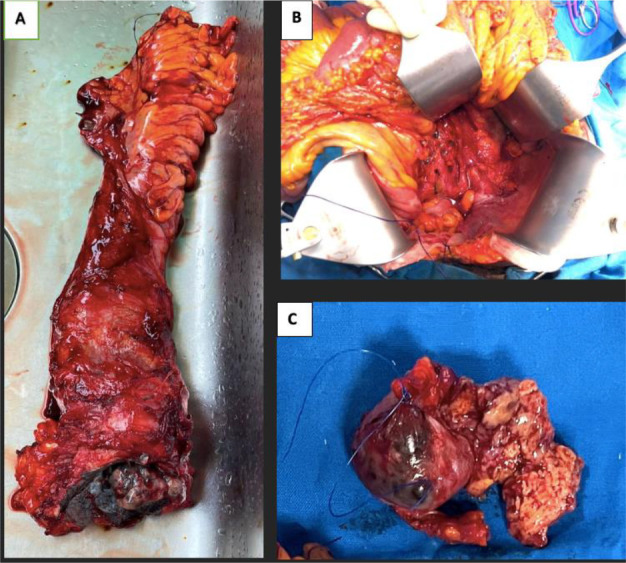
Pictures of the resected specimen. A: rectum and anus with a lesion in the anal canal. B: Shows the bed of black lymph nodes doted along the ventral vasculature extending proximally. C: Single right Inguinal lymph node (raptured during the dissection) specimen.

With persistent pain and anaemia (haemoglobin of 5.5g/dl), and no recommended neo-adjuvant therapy for this type of tumour, the decision was made to resect the tumour. She had an open abdominoperineal resection of the rectum with total mesorectal excision (and an end colostomy) and cherry-picking of lymph nodes proximally along the central vasculature as far as was feasible, and lymphadenectomy for a large right inguinal lymph node (Figure 3). The iliac chain of lymph nodes was not dissected.

Her carcinoembryonic antigen (CEA) was 1.6 nanograms/millilitre (ng/ml)

**Figure 4:**
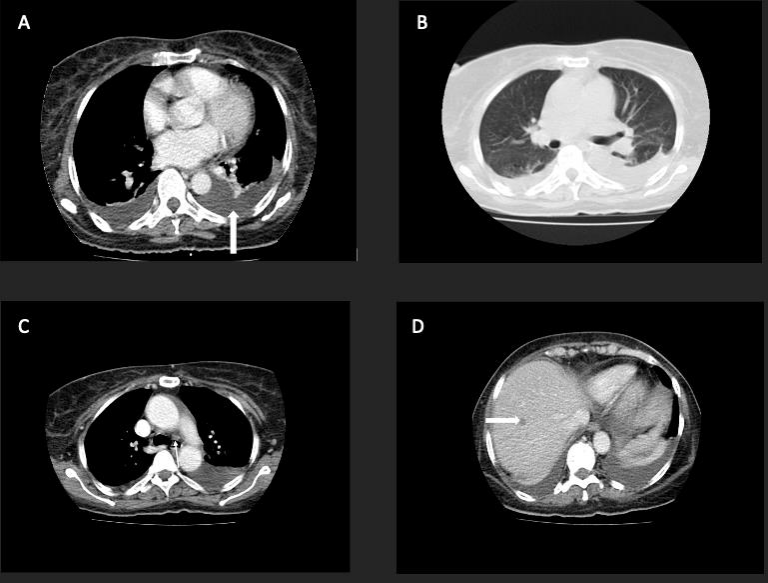
Contrast-enhanced axial computer tomography (CT) scan of the chest of the index patient. A: The mediastinal window shows moderate bilateral pleural effusion (left pleural effusion white arrow). B: No lung nodules are seen in the lung window. C: A small prominent pre-tracheal lymph node ∼ 6mm short axis dimension (arrowed), (mediastinal window), and D: Axial projection shows a small, well-defined non-enhancing hypodense lesion in the liver measures 9.3mm (arrowed)

Computed tomography of the chest (two weeks post-operative) showed a bilateral moderate pleural effusion with a central mediastinal lymph node concerning metastasis. There were no lung nodules. A new well-defined subcentimeter hypodense lesion was also noted in the liver, raising concerns for metastasis (Figure 4).

Histopathology of the gross specimen confirmed a poorly differentiated mucosal malignant melanoma, with a tumour invading the muscularis propria into the pericolorectal tissues but free distal and circumferential tumour margins. Thirty-nine of the 43 lymph nodes had scattered malignant cells. There was a perineural invasion. The tumour was staged at pT3 N2b. Immunohistochemistry staining was diffusely positive for S-100, suggesting a melanin-rich malignant melanoma. She had an uneventful recovery and was discharged on the seventh day after surgery. Further testing for possible immunotherapy-based adjuvant therapy was negative for programmed death-ligand 1 (PD-L1) protein, the PD-L1 expression being <1%. She was started on Temozolomide 150mg/m2, Days 1-5, in a 28-day cycle.The patient will have a scheduled follow-up to assess for new progression metastatic lesions, local recurrence, and overall survival.

### Ethical approval and Patient consent for publication:

Written and informed consent has been obtained from the patient to publish the case report and images in the journal

## Discussion

Mucosal melanomas arise from mucosal epithelial lining that contains melanocytes, commonly in the oral cavity, pharynx, vulvovaginal, anorectal, and urinary tract. Compared to melanomas from cutaneous sites, mucosal melanomas have a poor prognosis.^5^, ^6^ Mucosal melanomas frequently present mutations in the cell surface receptor tyrosine kinase activation (c-KIT), compared to cutaneous melanomas, which are associated with high BRAF mutations, rare in AMM.^7^

Symptoms of AMM are nonspecific, as in common anorectal disorders such as haemorrhoids, polyps, or other benign anorectal pathology, and may be the main reason for delays in diagnosis. When tumours become more extensive, they may cause an anal mass with pain, constipation, and bleeding, and further investigations make an incidental diagnosis. The index patient had a five-year history of symptoms suggestive of haemorrhoids and was managed for it over the last two years and was only referred for further investigations when symptoms worsened.

Histology and immunohistochemistry are used to confirm the diagnosis of AMM, and head, thorax, abdomen, and pelvis CT scan help in the pathology staging. A complete colonoscopy picks up synchronous lesions, and in the index patient, a colonoscopy showed a benign adenomatous polyp in the caecum.

Up to a third of patients already have metastasis at the time of diagnosis due to the aggressive nature of the lesion and the late detection.^8^ **The** patient had a tumour invading the muscularis propria into the pericolorectal tissues with perineural invasion. She grossly had a tumour in the inguinal lymph nodes and a pre-tracheal lymph node per CT scan. AMM with perineural invasion is associated with recurrence.^9^, ^10^ This patient has a high risk of tumour recurrence as histology showed perineural invasion.

There are no clear guidelines for treating AMM, and outcomes for treatment have not changed over the years. Surgery has been reported to be the most effective treatment for anorectal melanoma.^11^ Surgical options include a wide local excision (WLE) or abdominoperineal resection (APR) of the rectum. Some studies have shown improved overall survival for patients following APR compared with WLE. However, in other studies, the radicality of an APR has not demonstrated improved survival compared to WLE.^12^, ^13^ Wide local excision minimizes the morbidity associated with surgery but is not considered if there appears to be sphincter involvement. In this report, an APR was performed due to the anal mass impairing patient's sitting and faecal incontinence, suggesting anal sphincter involvement.

In **this** patient, cherry-picking of lymph nodes as far proximally as was feasible was done without increasing intra-operative morbidity. An extensive lymph node dissection of the central vasculature may have increased morbidity. The iliac chain of lymph nodes was not dissected since it may have increased intra-operative morbidity. The role of extensive lymphadenectomy is still unclear for AMM, and there is no optimal primary nodal management strategy. Studies have indicated that patients with or without lymph node metastasis in the mesorectum had a similar prognosis for local or distant disease recurrence and survival.^9^

Malignant melanoma appears as a cytoplasmic brown to black coloured pigment on histopathology, and atypical epidermoid cells are seen adjacent to the focus of the malignant tumour. In the absence of these features, the diagnosis of melanoma depends on immunohistochemistry. Melanoma can resemble different tumours, including carcinomas, neuroendocrine tumours, sarcomas, lymphomas, and germ cell tumours. Immunohistochemical staining for melanocytic markers of differentiation is often employed in diagnosing melanoma. Melanoma cells are positive for S-100, Human Melanoma Black-45 (HMB-45), vimentin, melanoma antigen (Melan-A), tyrosinase, and microphthalmia transcription factor (MITF).^14^, ^15^ Melan-A, HMB-45, and tyrosinase show diminishing sensitivity with advancing-stage disease and cannot distinguish malignant from non-malignant melanocytic lesions. S-100 has much greater sensitivity in desmoplastic malignant melanoma.^15^ Immunohistochemistry in the index patient was strongly positive for S-100.

Non-surgical treatment is varied and includes radiotherapy, chemotherapy, and targeted therapies used alone or in various combinations. Immunotherapy with alfa interferon, brachytherapy with 117-Caesium, and chemotherapy with dacarbazine, vincristine, nimustine hydrochloride, and targeted drugs such as ipilimumab are recent advances in the treatment of AMM.^16^, ^17^ The average survival has not exceeded 15-20 months, with no improvement with adjuvant chemoradiation or immunotherapy currently used for cutaneous melanomas.^18^ Significant differences at the molecular level exist between cutaneous and anorectal melanomas, evident in the expression of BRAF gene mutation and the variations in molecular pathogenesis and consequent therapeutic implications.^18^

Despite the inconclusive evidence of the role of immunotherapy, PD-L1 testing was done to commence immunotherapy for the index patient, but a <1% expression indicated she was not going to benefit from immunotherapy. Another option in her line of management, testing for BRAF V 600 mutation, is not available in Ghana and is very expensive for most patients. The recommended targeted therapy regimens, such as Dabrafenib and Trametinib, are unavailable for patients with these mutations. Therefore, the decision to manage palliatively with Temozolomide, a suggested cytotoxic regimen for metastatic malignant melanoma recommended by the National Comprehensive Cancer Network and available in Ghana.

The role of radiotherapy in treating AMM is unclear and is used more effectively in the palliative setting. No consensus currently exists on adequate systemic therapy for anorectal melanoma. Ongoing research will clarify the role of immunotherapy in the neoadjuvant and adjuvant settings in the management of anorectal melanoma. The patient is having adjuvant therapy, and the prognosis of her condition is monitored.

## Conclusion

AMM is rare, with a poor prognosis. Guidelines for the treatment of AMM are unclear, especially in the aspect of neoadjuvant or adjuvant therapies. Surgical resection with free margins manages the local disease, and other systemic therapies, including immunotherapy, may improve prognosis.
